# SNPnexus: an enhanced web platform for large-scale and multi-sample variant analysis (2025 update)

**DOI:** 10.1093/nar/gkag461

**Published:** 2026-05-11

**Authors:** Jorge Oscanoa, Qianqian Zhu, Emanuela Gadaleta, Claude Chelala

**Affiliations:** Centre for Biomarkers and Biotherapeutics, Barts Cancer Institute, Queen Mary University of London, London, EC1M 6BQ, United Kingdom; Centre for Biomarkers and Biotherapeutics, Barts Cancer Institute, Queen Mary University of London, London, EC1M 6BQ, United Kingdom; Centre for Biomarkers and Biotherapeutics, Barts Cancer Institute, Queen Mary University of London, London, EC1M 6BQ, United Kingdom; Centre for Biomarkers and Biotherapeutics, Barts Cancer Institute, Queen Mary University of London, London, EC1M 6BQ, United Kingdom

## Abstract

SNPnexus is a long-standing web-based platform for the functional annotation and prioritization of genetic variants. Since its previous release, SNPnexus has undergone substantial backend re-engineering resulting in major performance improvements and a complete restructuring of the underlying datasets. A redesigned interface now enables larger queries and multi-sample analysis enabling comparative workflows such as identifying shared pathogenic variants in disease cohorts and divergent mutations in cancer evolution studies. SNPnexus increased its capacity to 150 000 variants per query and introduced pre-annotation filters that allow users to restrict analyses to selected genes or genomic regions, improving efficiency while enabling targeted interrogation of high–priority targets. SNPnexus integrates updated and expanded annotations across both GRCh37 and GRCh38, covering genomic consequences, *in silico* pathogenicity predictions, population allele frequencies, evolutionary conservation, regulatory elements, biological pathways, and clinical associations. The refreshed result interface provides interactive visualizations for single–sample and cohort–level outputs, together with advanced filtering and export options. Optional user accounts now support query history and real–time job monitoring while preserving full, unregistered access for all users. SNPnexus remains free and open to all users without login requirements at https://snpnexus.org/.

## Graphical Abstract

**Graphical Abstract:**
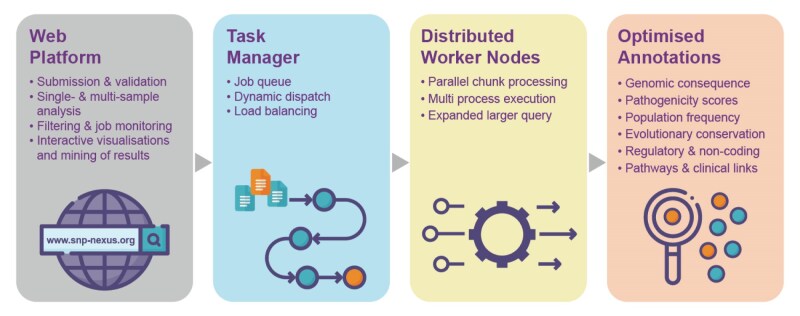

## Introduction

The widespread availability of next generation sequencing across research and clinical settings continues to generate vast quantities of genomic variation data, creating an urgent need for efficient tools capable of transforming raw variant lists into biologically interpretable insights. Despite extensive progress in computational genomics, the functional annotation and prioritization of variants remain major bottlenecks in precision medicine, cancer genomics, and population-scale studies, where researchers must integrate heterogeneous datasets, pathogenicity predictions, biological context, and clinical associations.

SNPnexus was originally developed to address this challenge by providing a high-throughput, user-friendly platform for variant annotation, enabling researchers to interrogate genomic variation without requiring local computational infrastructure [1–4].

Over successive development cycles, SNPnexus has been refined in response to community feedback and evolving scientific needs, establishing itself as a widely used platform for functional interpretation of genetic variants.

The rapid growth of cohort–based and longitudinal sequencing studies has created a strong need for tools that support comparative analysis across multiple samples, which is a capability not addressed by earlier versions of SNPnexus. In this release, we present the fifth major upgrade of the SNPnexus web platform introducing a new multi-sample analysis framework that allows simultaneous processing and intersection of up to six sample sequencing files. This development enables workflows such as identifying shared pathogenic variants and characterizing divergent mutations in cancer evolution studies, significantly reducing the manual bioinformatic burden previously required for such analyses.

Alongside this, extensive backend refactoring and optimization have increased the platform capacity to 150 000 variants per query, overcoming previous high-throughput constraints. Newly introduced pre-annotation filters further allow users to restrict processing to high-priority genes or genomic regions, enabling targeted analysis that avoid the computational overhead of processing whole-genome variant sets or non-informative regions. A comprehensive restructuring of underlying datasets ensures improved consistency, broader annotation coverage, and enhanced performance across both GRCh37 and GRCh38 reference assemblies.

Together, these advances position the 2025 release of SNPnexus as a significantly expanded and modernized platform designed to meet the evolving needs of the genomics community. The updated web server provides scalable architecture, extensive functional annotations, multi–sample comparative workflows, and improved user experience, ensuring that SNPnexus remains a cutting-edge and sustainable resource in the variant annotation landscape.

## Data sources and integration

SNPnexus aggregates and integrates a broad collection of datasets, all harmonized into platform-optimized formats to ensure high-speed annotation. The 2025 update includes a comprehensive audit of all underlying datasets, with outdated resources removed and new high-impact annotations incorporated. The platform maintains full support for both GRCh37 and GRCh38 human reference assemblies, employing Liftover where necessary to guarantee equivalent coverage across builds.

SNPnexus organizes its annotations into several major categories:

Genomic consequence: functional impact predictions based on transcript-level allele substitution, using Ensembl [5], NCBI RefSeq [6], and the Consensus CDS (CCDS) [7] models, and reported HGVS nomenclature [8].
*In silico p*athogenicity predictions: scores from SIFT [9], PolyPhen2 [10], REVEL [11], AlphaMissense [12], CADD [13], ReMM [14], and Jarvis [15] for missense and non-coding single nucleotide variants (SNVs), with established pathogenicity thresholds in Supplementary Table S1.
Population allele frequency: variant prevalence across global populations using Allele Frequency Aggregator [16], 1000 Genomes Project [17], and the Genome Aggregation Database [18].
Evolutionary conservation: PhastCons [19] (based on 100 vertebrates alignments) and the Genomic Evolutionary Rate Profiling [20] scores (90 mammals alignment) to highlight constraints across evolutionary histories.
Non-coding RNAs and regulatory elements: data from miRBase [21], RNAcentral [22] and TarBase [23], as well as regulatory elements from the Ensembl Regulatory Build [24] and Encode [25].
Biological context and pathway analysis: tissue-specific expression from the Genotype Tissue Expression (GTEx) [26], Reactome [27] pathway enrichment via Fisher’s exact test, and KEGG [28] functional analysis through the GSEApy library [29].
Clinical and phenotypic association: cross-referencing with ClinVar [30], COSMIC [31], and the GWAS Catalog [32] to link variants to known diseases and traits.

The modular architecture of the annotation layer enables rapid incorporation of new resources defined by genomic coordinates or gene symbols. To ensure results remain current, clinical and phenotypic association datasets are updated quarterly, while all other datasets are refreshed every six month. A complete, versioned list of all integrated datasets is provided in Supplementary Table S2.

## Pipeline and architecture

The 2025 release incorporates a fully redesigned system architecture structured around four layers to support higher performance, scalability, and multi–sample workflows.

1. Web platform: The web layer handles user authentication, query validation, submission, and real-time job monitoring. It also provides interfaces for browsing results, accessing interactive visualizations, and applying advanced filters.
2. Task manager: A dedicated task management service orchestrates the job queue, dynamically dispatching user queries to available worker nodes for optimal load balancing and reduced processing times.
3. Distributed worker nodes: Each node runs a Core Annotation Engine (CAE) using a multi-process strategy. For large submissions (>10 000 variants), the CAE segments input data into parallelizable chunks to minimize latency and support the expanded limit of 150 000 variants per query.
4. Optimized annotations repository: The backend employs a customized indexed annotation repository designed for rapid retrieval by the CAE. This optimized data layer underpins the considerable performance improvements introduced in this version.

**Figure 1.**
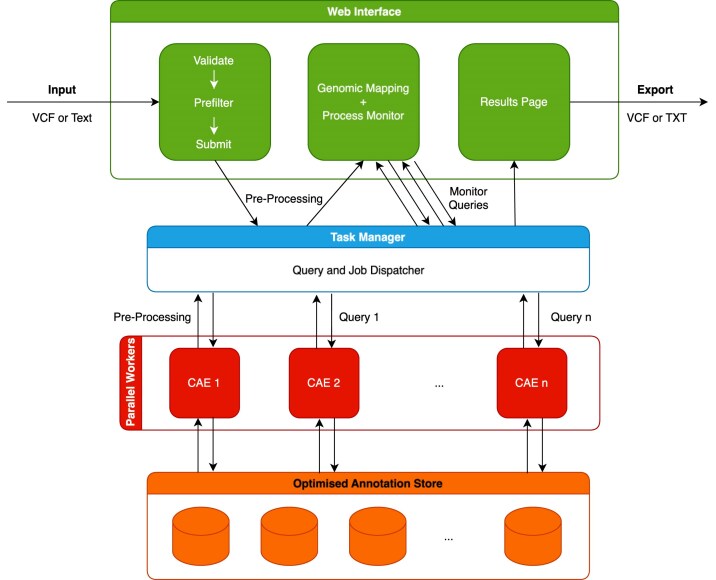
Overview of SNPnexus renewed pipeline and system architecture.

An overview of the complete system workflow and architecture is provided in Fig. 1.

User submissions are first validated with an optional pre–annotation filtering step applied via the web interface. Jobs are then dispatched by a central task manager to distributed CAE worker nodes for parallel processing. Annotation results are retrieved from an optimized annotation store and returned through the results interface, supporting interactive visualization and export for both single– and multi–sample analyses.

## Results, visualization, and filtering

The refreshed SNPnexus results interface is optimized to support both single- and multi-sample exploration, providing an intuitive environment for navigating large variant datasets. For multi–sample analyses, each sample is presented in a dedicated tab containing sample-specific visualizations, including genomic consequence summaries, *in silico* prediction distributions, Reactome Reacfoam diagrams, and KEGG pathway enrichment results. These outputs are accompanied by interactive result tables that support keyword-based searching and export to TSV or VCF formats. Results are retained on the platform for 5 days, during which users may download the complete set of annotations as a consolidated ZIP archive.

A new section provides cohort-level visualizations across all submitted samples, summarizing key mutational features such as the most frequently mutated genes, overall variant-type distributions, an oncoplot of non-synonymous mutations in at least two samples, and a lollipop plot highlighting recurrent mutation hotspots within individual genes. These visual summaries aid users in identifying shared pathogenic variants, divergent mutational patterns, and other biologically meaningful trends across cohorts.

Advance filtering tools allow users to refine results based on mutation novelty (known versus novel), genomic consequence categories (e.g. non-synonymous, synonymous, and intronic), global allele frequency thresholds, or regions overlapping specific genes or gene sets. For multi-sample analyses, comparative filtering enables isolation of variants shared across certain samples or those unique to individual samples. Filters apply globally across the interface, dynamically updating tables and visualizations to reflect the selected constraints in real time.

### Use case

The expanded SNPnexus framework supports submission of up to six samples per query, enabling integrated cohort-level analysis alongside stratified patient-level comparisons.

To illustrate these capabilities, we generated a synthetic dataset comprising variant profiles from six breast cancer patients, comprising three hormone receptor-positive cases (P1–P3) and three triple negative cases (P4–P6). The dataset was curated to include both shared and subtype-specific variants, allowing evaluation of comparative analytical workflows. The results from this use case can be browsed in https://snpnexus.org/results/nar2026/.

**Figure 2.**
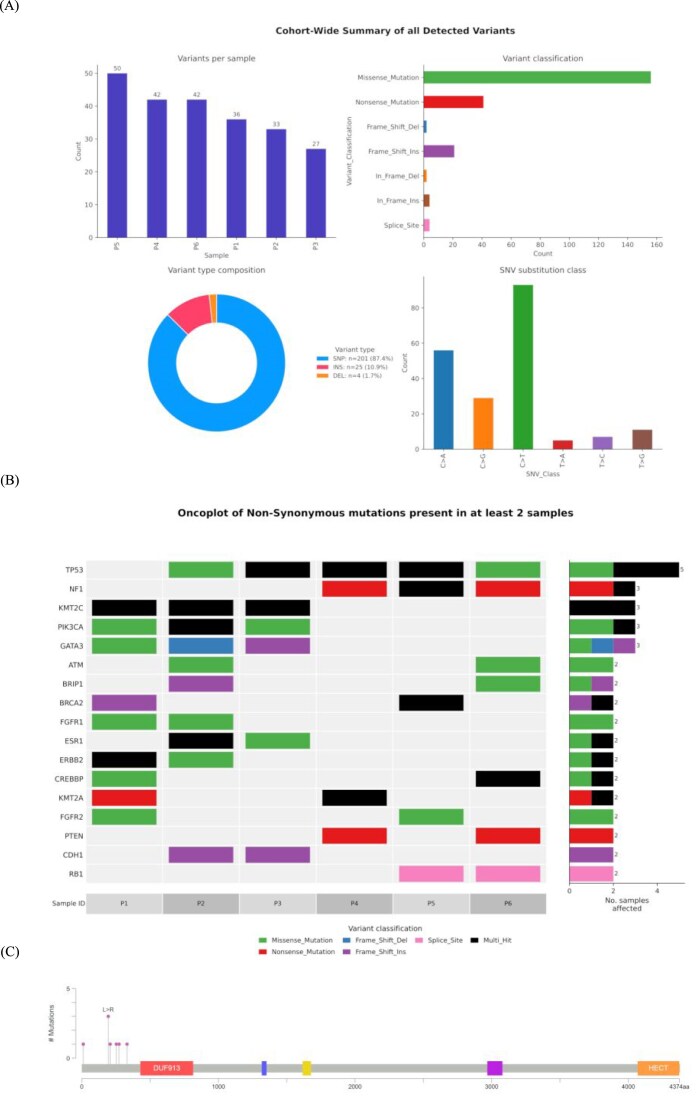
Cohort-level visual summaries obtained for the use case. (**A**) Cohort-wide summary of variants. (**B**) Non-synonymous mutations in recurrently mutated genes. (**C**) Gene-level distribution of TP53 variants, highlighting recurrence and positional clustering.

Cohort-level summaries provide a descriptive overview of variant classes (Fig. 2A). Consistent with published literature, *TP53* is recurrently mutated in 83% of the cohort and is present in both subtypes. Other genes, such as *BRCA2*, are less-frequently mutated in this cohort and do not show clear subtype association.

Distinct subtype-specific patterns are evident for *GATA3, KMT2C*, and *PIK3CA*, which are mutated in all hormone receptor-positive samples, while *NF1, RB1*, and *PTEN* variants are restricted to the triple-negative subgroup. Gene-level interrogation further highlights recurrent mutational patterns and positional clustering within individual genes (Fig. 2B and C), supporting downstream biological interpretation.

## Discussion and future plans

Over the years, SNPnexus has evolved in response to community feedback and changing analytical demands, with an emphasis on maintaining usability while integrating new functionalities.

The enhancements introduced in the 2025 release position SNPnexus as a modern, scalable platform for functional variant annotation, addressing the growing need for cohort–based and comparative analyses.

Several established tools provide functional annotation of genomic variants. Web–based services such as wAnnovar [33] or the Ensembl Variant Effect Predictor [34] offer widely used annotation pipelines, while platforms such as VarSome [35] and DECIPHER [36] provide interactive interfaces that integrate variant annotation with clinical and phenotypic context. Other resources such as SnpXplorer [37] focus on exploration of SNP–association data with more limited annotation breadth, and OpenCravat [38] provides an extensible framework supporting a wide range of annotations and custom modules.

While these tools address overlapping aspects of variant interpretation, they differ in scope, scalability, and support for comparative analysis. To our knowledge, SNPnexus is currently the only fully web–based platform that combines large–scale variant annotation with native multi–sample comparative workflows, enabling direct interrogation of shared and divergent mutational patterns across cohorts without requiring local installation or command–line configuration. A detailed comparison of SNPnexus with existing tools, including supported annotations, query capacity, and analytical features, is provided in Supplementary Table S3.

To validate performance gains, we benchmarked the current SNPnexus version against its predecessor using varied input sets and numbers of worker nodes (Supplementary Table S4). Using the Ensembl genomic consequence annotation as the benchmarking metric, the updated system consistently outperformed the previous version by a factor of 1.5–4.8, with the highest efficiency gains observed for large datasets. Consequently, we expanded the user query limit to 150 000 variants per submission and established a maximum of six samples per query, balancing performance with equitable resource allocation while maintaining broad accessibility. Although these thresholds support most high–throughput applications, the redesigned architecture provides a scalable foundation for future growth. Ongoing development is focused on accommodating even larger datasets and expanded cohorts in future releases. The introduction of pre-annotation filters further extends the utility of the platform, allowing targeted analysis of high-priority loci and reducing the computational burden associated with whole–genome variant lists.

Future developments will focus on three core development objectives that will guide future iterations of SNPnexus. First, a RESTful API is under active development to provide programmatic accessibility, enabling seamless integration of the SNPnexus CAE directly into automated pipelines and high-throughput workflows commonly used by bioinformaticians. Second, upcoming releases will focus on clinical decision support, aiming to transition from purely functional annotation towards standardized clinical interpretation. This will include automated implementation of ACMG/AMP and ACGS guidelines [39, 40] to assist with reliable classification of clinical significance and facilitate translational research and diagnostic applications. Finally, the modularity of the newly redesigned architecture supports extensibility and partnerships, enabling the platform to serve as a foundation for specialized genomic portals developed in collaboration with external institutions. Several such collaborations are already underway, and additional partnerships will be pursued to further expand the reach and impact of SNPnexus.

Together, these developments underscore SNPnexus’s commitment to sustainability, community responsiveness, and continued innovation. As genomic datasets grow in scale and complexity, SNPnexus is well–positioned to remain a central resource for comprehensive, high–performance variant annotation and interpretation.

## Supplementary Material

gkag461_Supplemental_Files

## Data Availability

The SNPnexus site is free to all users. It does not require registration, and is publicly available at https://www.snpnexus.org with a detailed user guide.

## References

[1] Chelala C, Khan A, Lemoine NR. SNPnexus: a web database for functional annotation of newly discovered and public domain single nucleotide polymorphisms. Bioinformatics. 2009;25:655–61. 10.1093/bioinformatics/btn653 19098027 PMC2647830

[2] Oscanoa J, Sivapalan L, Gadaleta E et al. SNPnexus: a web server for functional annotation of human genome sequence variation (2020 update). Nucleic Acids Res. 2020;48:W185–92. 10.1093/nar/gkaa420 32496546 PMC7319579

[3] Dayem Ullah AZ, Lemoine NR, Chelala C. SNPnexus: a web server for functional annotation of novel and publicly known genetic variants (2012 update). Nucleic Acids Res. 2012;40:W65–70. 10.1093/nar/gks364 22544707 PMC3394262

[4] Dayem Ullah AZ, Oscanoa J, Wang J et al. SNPnexus: assessing the functional relevance of genetic variation to facilitate the promise of precision medicine. Nucleic Acids Res. 2018;46:W109–13. 10.1093/nar/gky399 29757393 PMC6030955

[5] Dyer SC, Austine-Orimoloye O, Azov AG et al. Ensembl 2025. Nucleic Acids Res. 2025;53:D948–57. 10.1093/nar/gkae1071 39656687 PMC11701638

[6] Goldfarb T, Kodali VK, Pujar S et al. NCBI RefSeq: reference sequence standards through 25 years of curation and annotation. Nucleic Acids Res. 2025;53:D243–57. 10.1093/nar/gkae1038 39526381 PMC11701664

[7] Pujar S, O’Leary NA, Farrell CM et al. Consensus coding sequence (CCDS) database: a standardized set of human and mouse protein-coding regions supported by expert curation. Nucleic Acids Res. 2018;46:D221–8. 10.1093/nar/gkx1031 29126148 PMC5753299

[8] Hart RK, Fokkema I, DiStefano M et al. HGVS Nomenclature 2024: improvements to community engagement, usability, and computability. Genome Med. 2024;16:149. 10.1186/s13073-024-01421-5 39702242 PMC11660784

[9] Sim NL, Kumar P, Hu J et al. SIFT web server: predicting effects of amino acid substitutions on proteins. Nucleic Acids Res. 2012;40:W452–7. 10.1093/nar/gks539 22689647 PMC3394338

[10] Adzhubei IA, Schmidt S, Peshkin L et al. A method and server for predicting damaging missense mutations. Nat Methods. 2010;7:248–9. 10.1038/nmeth0410-248 20354512 PMC2855889

[11] Ioannidis NM, Rothstein JH, Pejaver V et al. REVEL: an ensemble method for predicting the pathogenicity of rare missense variants. Am J Hum Genet. 2016;99:877–85. 10.1016/j.ajhg.2016.08.016 27666373 PMC5065685

[12] Cheng J, Novati G, Pan J et al. Accurate proteome-wide missense variant effect prediction with AlphaMissense. Science. 2023;381:eadg7492. 10.1126/science.adg7492 37733863

[13] Schubach M, Maass T, Nazaretyan L et al. CADD v1.7: using protein language models, regulatory CNNs and other nucleotide-level scores to improve genome-wide variant predictions. Nucleic Acids Res. 2024;52:D1143–54. 10.1093/nar/gkad989 38183205 PMC10767851

[14] Schubach M, Nazaretyan L, Kircher M. The Regulatory Mendelian Mutation score for GRCh38. GigaScience. 2022;12:giad024. 10.1093/gigascience/giad024 37083939 PMC10120424

[15] Vitsios D, Dhindsa RS, Middleton L et al. Prioritizing non-coding regions based on human genomic constraint and sequence context with deep learning. Nat Commun. 2021;12:1504. 10.1038/s41467-021-21790-4 33686085 PMC7940646

[16] Phan L, Y.J. HZ, Qiang W et al. "ALFA: Allele Frequency Aggregator.". National Center for Biotechnology Information, U.S. National Library of Medicine, www.ncbi.nlm.nih.gov/snp/docs/gsr/alfa/, 10 March 2020.

[17] Genomes Project C, Auton A, Brooks LD et al. A global reference for human genetic variation. Nature. 2015;526:68–74. 10.1038/nature15393 26432245 PMC4750478

[18] Chen S, Francioli LC, Goodrich JK et al. A genomic mutational constraint map using variation in 76,156 human genomes. Nature. 2024;625:92–100. 10.1038/s41586-023-06045-0 38057664 PMC11629659

[19] Hubisz MJ, Pollard KS, Siepel A. PHAST and RPHAST: phylogenetic analysis with space/time models. Brief Bioinform. 2011;12:41–51. 10.1093/bib/bbq072 21278375 PMC3030812

[20] Davydov EV, Goode DL, Sirota M et al. Identifying a high fraction of the human genome to be under selective constraint using GERP++. PLoS Comput Biol. 2010;6:e1001025. 10.1371/journal.pcbi.1001025 21152010 PMC2996323

[21] Kozomara A, Birgaoanu M, Griffiths-Jones S. miRBase: from microRNA sequences to function. Nucleic Acids Res. 2019;47:D155–62. 10.1093/nar/gky1141 30423142 PMC6323917

[22] The RC . RNAcentral: a hub of information for non-coding RNA sequences. Nucleic Acids Res. 2019;47:D221–9. 10.1093/nar/gky1034 30395267 PMC6324050

[23] Skoufos G, Kakoulidis P, Tastsoglou S et al. TarBase-v9.0 extends experimentally supported miRNA–gene interactions to cell-types and virally encoded miRNAs. Nucleic Acids Res. 2024;52:D304–10. 10.1093/nar/gkad1071 37986224 PMC10767993

[24] Zerbino DR, Wilder SP, Johnson N et al. The ensembl regulatory build. Genome Biol. 2015;16:56. 10.1186/s13059-015-0621-5 25887522 PMC4407537

[25] Consortium EP . An integrated encyclopedia of DNA elements in the human genome. Nature. 2012;489:57–74. 10.1038/nature11247 22955616 PMC3439153

[26] Consortium GT . The Genotype-Tissue Expression (GTEx) project. Nat Genet. 2013;45:580–5. 10.1038/ng.2653 23715323 PMC4010069

[27] Milacic M, Beavers D, Conley P et al. The Reactome Pathway Knowledgebase 2024. Nucleic Acids Res. 2024;52:D672–8. 10.1093/nar/gkad1025 37941124 PMC10767911

[28] Kanehisa M, Furumichi M, Sato Y et al. KEGG: biological systems database as a model of the real world. Nucleic Acids Res. 2025;53:D672–7. 10.1093/nar/gkae909 39417505 PMC11701520

[29] Fang Z, Liu X, Peltz G. GSEApy: a comprehensive package for performing gene set enrichment analysis in Python. Bioinformatics. 2023;39:btac757. 10.1093/bioinformatics/btac757 36426870 PMC9805564

[30] Landrum MJ, Lee JM, Benson M et al. ClinVar: improving access to variant interpretations and supporting evidence. Nucleic Acids Res. 2018;46:D1062–7. 10.1093/nar/gkx1153 29165669 PMC5753237

[31] Sondka Z, Dhir NB, Carvalho-Silva D et al. COSMIC: a curated database of somatic variants and clinical data for cancer. Nucleic Acids Res. 2024;52:D1210–7. 10.1093/nar/gkad986 38183204 PMC10767972

[32] Cerezo M, Sollis E, Ji Y et al. The NHGRI-EBI GWAS Catalog: standards for reusability, sustainability and diversity. Nucleic Acids Res. 2025;53:D998–D1005. 10.1093/nar/gkae1070 39530240 PMC11701593

[33] Chang X, Wang K. wANNOVAR: annotating genetic variants for personal genomes via the web. J Med Genet. 2012;49:433–6. 10.1136/jmedgenet-2012-100918 22717648 PMC3556337

[34] McLaren W, Gil L, Hunt SE et al. The Ensembl Variant Effect Predictor. Genome Biol. 2016;17:122. 10.1186/s13059-016-0974-4 27268795 PMC4893825

[35] Kopanos C, Tsiolkas V, Kouris A et al. VarSome: the human genomic variant search engine. Bioinformatics. 2019;35:1978–80. 10.1093/bioinformatics/bty897 30376034 PMC6546127

[36] Foreman J, Perrett D, Mazaika E et al. DECIPHER: improving genetic diagnosis through dynamic integration of genomic and clinical data. Annu Rev Genomics Hum Genet. 2023;24:151–76. 10.1146/annurev-genom-102822-100509 37285546 PMC7615097

[37] Tesi N, van der Lee S, Hulsman M et al. snpXplorer: a web application to explore human SNP-associations and annotate SNP-sets. Nucleic Acids Res. 2021;49:W603–12. 10.1093/nar/gkab410 34048563 PMC8262737

[38] Pagel KA, Kim R, Moad K et al. Integrated informatics analysis of cancer-related variants. JCO Clin Cancer Inform. 2020;4:310–7. 10.1200/CCI.19.00132 32228266 PMC7113103

[39] Burghel GJ, Mason J, Baker K et al. Association for Clinical Genomic Science (ACGS) guidelines for the classification of oncogenicity of somatic variants in cancer: recommendations by the UK somatic variant interpretation group (SVIG-UK). J Med Genet. 2026;63:147–56. 10.1136/jmg-2025-111046 41339070 PMC13018753

[40] Richards S, Aziz N, Bale S et al. Standards and guidelines for the interpretation of sequence variants: a joint consensus recommendation of the American College of Medical Genetics and Genomics and the Association for Molecular Pathology. Genet Med. 2015;17:405–24. 10.1038/gim.2015.30 25741868 PMC4544753

